# Supplementary material for: Modeling glioblastoma heterogeneity as a dynamic network of cell states
Source: Mol Syst Biol. 2021 Sep 16;17(9):e10105. doi: 10.15252/msb.202010105 (PMC8444284; doi:10.15252/msb.202010105)
Supplement: Supplementary file 6 — Source Data for Figure 5 [file MSB-17-e10105-s004.zip › Figure5A_sourcedata/GSEA_3017/nosigdb_stateD.GseaPreranked.1622795010397/gsea_report_for_na_neg_1622795010397.html]

Report for na\_neg 1622795010397 [GSEA]

| GS  follow link to MSigDB | GS DETAILS | SIZE | ES | NES | NOM p-val | FDR q-val | FWER p-val | RANK AT MAX | LEADING EDGE || 1 | FELDMAN\_2020\_G1 | Details ... | 88 | -0.65 | -3.51 | 0.000 | 0.000 | 0.000 | 180 | tags=58%, list=14%, signal=63% |
| 2 | LARSSON\_STATE\_4 | Details ... | 66 | -0.68 | -3.46 | 0.000 | 0.000 | 0.000 | 189 | tags=61%, list=14%, signal=67% |
| 3 | DALMO\_BARCODENUMBER\_UNPUB | Details ... | 105 | -0.62 | -3.38 | 0.000 | 0.000 | 0.000 | 210 | tags=56%, list=16%, signal=62% |
| 4 | DOROSZKO\_U3013MG\_CLUSTER\_2 | Details ... | 142 | -0.56 | -3.20 | 0.000 | 0.000 | 0.000 | 327 | tags=61%, list=25%, signal=73% |
| 5 | FELDMAN\_2020\_S-G2 | Details ... | 165 | -0.56 | -3.19 | 0.000 | 0.000 | 0.000 | 410 | tags=67%, list=31%, signal=85% |
| 6 | RICHARDS\_ZHONG\_NATURE\_2018\_NPCS\_UPREG | Details ... | 85 | -0.59 | -3.07 | 0.000 | 0.000 | 0.000 | 180 | tags=51%, list=14%, signal=55% |
| 7 | DOROSZKO\_U3180MG\_CLUSTER\_3 | Details ... | 232 | -0.50 | -2.99 | 0.000 | 0.000 | 0.000 | 480 | tags=68%, list=36%, signal=88% |
| 8 | IVY\_CT | Details ... | 94 | -0.54 | -2.95 | 0.000 | 0.000 | 0.000 | 288 | tags=56%, list=22%, signal=67% |
| 9 | FELDMAN\_2020\_G2-M | Details ... | 135 | -0.52 | -2.93 | 0.000 | 0.000 | 0.000 | 331 | tags=57%, list=25%, signal=68% |
| 10 | FELDMAN\_2020\_M-EARLYG1 | Details ... | 40 | -0.65 | -2.93 | 0.000 | 0.000 | 0.000 | 133 | tags=50%, list=10%, signal=54% |
| 11 | DOROSZKO\_U3054MG\_CLUSTER\_4 | Details ... | 273 | -0.48 | -2.85 | 0.000 | 0.000 | 0.000 | 514 | tags=67%, list=39%, signal=87% |
| 12 | FELDMAN\_2020\_NEURAL G0 | Details ... | 134 | -0.50 | -2.85 | 0.000 | 0.000 | 0.000 | 223 | tags=44%, list=17%, signal=48% |
| 13 | SEURAT\_CELLCYCLE | Details ... | 68 | -0.55 | -2.80 | 0.000 | 0.000 | 0.000 | 206 | tags=51%, list=16%, signal=58% |
| 14 | RICHARDS\_NEFTEL\_CELL\_2019\_G1\_S | Details ... | 24 | -0.68 | -2.76 | 0.000 | 0.000 | 0.000 | 223 | tags=71%, list=17%, signal=84% |
| 15 | NB\_KI\_CLUSTER5 | Details ... | 56 | -0.56 | -2.75 | 0.000 | 0.000 | 0.000 | 287 | tags=59%, list=22%, signal=72% |
| 16 | RICHARDS\_NOWAKOWSKI\_SCIENCE\_2017\_MGE\_IPC2\_UPREG | Details ... | 251 | -0.46 | -2.71 | 0.000 | 0.000 | 0.000 | 534 | tags=67%, list=40%, signal=92% |
| 17 | SUVA\_G1S\_PMID\_31327527 | Details ... | 24 | -0.68 | -2.70 | 0.000 | 0.000 | 0.000 | 223 | tags=71%, list=17%, signal=84% |
| 18 | GAROFANO\_IAVARONE\_PPR\_SUBTYPE | Details ... | 173 | -0.46 | -2.66 | 0.000 | 0.000 | 0.000 | 422 | tags=58%, list=32%, signal=74% |
| 19 | FELDMAN\_2020\_LATEG1 | Details ... | 47 | -0.56 | -2.65 | 0.000 | 0.000 | 0.000 | 227 | tags=51%, list=17%, signal=59% |
| 20 | PARADA\_T1\_UP\_26461091 | Details ... | 29 | -0.61 | -2.61 | 0.000 | 0.000 | 0.000 | 139 | tags=52%, list=11%, signal=57% |
| 21 | RICHARDS\_NEFTEL\_CELL\_2019\_AC |  | 26 | -0.64 | -2.60 | 0.000 | 0.000 | 0.000 | 195 | tags=58%, list=15%, signal=66% |
| 22 | SUVA\_AC\_PMID\_31327527 |  | 26 | -0.64 | -2.59 | 0.000 | 0.000 | 0.000 | 195 | tags=58%, list=15%, signal=66% |
| 23 | RICHARDS\_NOWAKOWSKI\_SCIENCE\_2017\_MGE\_DIV\_UPREG |  | 257 | -0.43 | -2.57 | 0.000 | 0.000 | 0.000 | 521 | tags=63%, list=39%, signal=84% |
| 24 | GANDAL\_AUTISM\_UP\_PMID29439242 |  | 22 | -0.65 | -2.49 | 0.000 | 0.000 | 0.000 | 108 | tags=50%, list=8%, signal=54% |
| 25 | LARSSON\_STATE\_6 |  | 39 | -0.54 | -2.46 | 0.000 | 0.000 | 0.000 | 213 | tags=49%, list=16%, signal=56% |
| 26 | FELDMAN\_2020\_S |  | 76 | -0.46 | -2.42 | 0.000 | 0.000 | 0.000 | 470 | tags=68%, list=36%, signal=100% |
| 27 | RICHARDS\_NOWAKOWSKI\_SCIENCE\_2017\_IPC\_DIV2\_UPREG |  | 320 | -0.40 | -2.39 | 0.000 | 0.000 | 0.000 | 425 | tags=50%, list=32%, signal=56% |
| 28 | GANDAL\_SCHIZOPHRENIA\_UP\_PMID29439242 |  | 37 | -0.52 | -2.35 | 0.000 | 0.000 | 0.001 | 215 | tags=49%, list=16%, signal=56% |
| 29 | GAROFANO\_IAVARONE\_LIST\_OF\_NEURAL\_PROGENITOR\_MARKER\_GENES |  | 42 | -0.51 | -2.34 | 0.000 | 0.000 | 0.001 | 288 | tags=55%, list=22%, signal=68% |
| 30 | RICHARDS\_NOWAKOWSKI\_SCIENCE\_2017\_RG\_EARLY\_UPREG |  | 154 | -0.41 | -2.32 | 0.000 | 0.000 | 0.002 | 242 | tags=36%, list=18%, signal=39% |
| 31 | RICHARDS\_NOWAKOWSKI\_SCIENCE\_2017\_IPC\_DIV1\_UPREG |  | 270 | -0.39 | -2.32 | 0.000 | 0.000 | 0.002 | 424 | tags=50%, list=32%, signal=59% |
| 32 | RICHARDS\_NOWAKOWSKI\_SCIENCE\_2017\_MGE\_IPC1\_UPREG |  | 227 | -0.40 | -2.32 | 0.000 | 0.000 | 0.002 | 327 | tags=41%, list=25%, signal=46% |
| 33 | NB\_KI\_CLUSTER18 |  | 31 | -0.52 | -2.24 | 0.000 | 0.000 | 0.011 | 195 | tags=39%, list=15%, signal=44% |
| 34 | RICHARDS\_NEFTEL\_CELL\_2019\_G2\_M |  | 31 | -0.50 | -2.21 | 0.000 | 0.000 | 0.015 | 240 | tags=52%, list=18%, signal=62% |
| 35 | RICHARDS\_NOWAKOWSKI\_SCIENCE\_2017\_U2\_UPREG |  | 435 | -0.35 | -2.15 | 0.000 | 0.001 | 0.033 | 350 | tags=38%, list=26%, signal=35% |
| 36 | SUVA\_G2M\_PMID\_31327527 |  | 31 | -0.50 | -2.15 | 0.000 | 0.001 | 0.034 | 240 | tags=52%, list=18%, signal=62% |
| 37 | ALLEN\_113\_ASTROL1FGFR3FOS |  | 17 | -0.59 | -2.10 | 0.000 | 0.002 | 0.058 | 119 | tags=41%, list=9%, signal=45% |
| 38 | OLSEN\_MESENCHYMAL |  | 29 | -0.48 | -2.05 | 0.000 | 0.002 | 0.092 | 113 | tags=31%, list=9%, signal=33% |
| 39 | PARADA\_T2\_UP\_26461091 |  | 25 | -0.50 | -2.03 | 0.002 | 0.003 | 0.112 | 165 | tags=44%, list=12%, signal=49% |
| 40 | RICHARDS\_CAHOY\_JNEUROSCI\_2016\_CULTURED\_ASTROGLIA |  | 282 | -0.34 | -2.01 | 0.000 | 0.003 | 0.134 | 433 | tags=47%, list=33%, signal=55% |
| 41 | OUTERRADIALGLIA |  | 22 | -0.51 | -2.00 | 0.005 | 0.003 | 0.139 | 418 | tags=68%, list=32%, signal=98% |
| 42 | POLLEN\_OUTERRADIALGLIA\_PMID\_26406371 |  | 22 | -0.51 | -1.99 | 0.001 | 0.004 | 0.163 | 418 | tags=68%, list=32%, signal=98% |
| 43 | RICHARDS\_NOWAKOWSKI\_SCIENCE\_2017\_RG\_DIV2\_UPREG |  | 427 | -0.33 | -1.96 | 0.000 | 0.005 | 0.208 | 443 | tags=45%, list=34%, signal=46% |
| 44 | SINGH\_SOX2\_UPREGULATED\_TRANSCRIPTS\_PMID\_28122245 |  | 121 | -0.35 | -1.96 | 0.000 | 0.005 | 0.216 | 407 | tags=45%, list=31%, signal=60% |
| 45 | SUVA\_MES1\_PMID\_31327527 |  | 18 | -0.54 | -1.95 | 0.001 | 0.006 | 0.239 | 159 | tags=44%, list=12%, signal=50% |
| 46 | RICHARDS\_CAHOY\_JNEUROSCI\_2010\_ASTRO\_YOUNG |  | 191 | -0.33 | -1.94 | 0.000 | 0.006 | 0.245 | 422 | tags=48%, list=32%, signal=60% |
| 47 | RICHARDS\_NEFTEL\_CELL\_2019\_MES1 |  | 18 | -0.54 | -1.93 | 0.005 | 0.006 | 0.276 | 159 | tags=44%, list=12%, signal=50% |
| 48 | OLSEN\_SCPLIKE |  | 37 | -0.42 | -1.91 | 0.007 | 0.008 | 0.324 | 237 | tags=38%, list=18%, signal=45% |
| 49 | RICHARDS\_ZHONG\_NATURE\_2018\_ASTROCYTES\_UPREG |  | 93 | -0.35 | -1.86 | 0.000 | 0.011 | 0.445 | 280 | tags=38%, list=21%, signal=44% |
| 50 | TISIG\_PMID\_32649888 |  | 21 | -0.49 | -1.86 | 0.010 | 0.011 | 0.452 | 304 | tags=57%, list=23%, signal=73% |
| 51 | DOROSZKO\_U3054MG\_CLUSTER\_2 |  | 62 | -0.37 | -1.84 | 0.004 | 0.013 | 0.515 | 296 | tags=40%, list=22%, signal=50% |
| 52 | GANDAL\_ALCOHOLADDICTION\_UP\_PMID29439242 |  | 24 | -0.46 | -1.80 | 0.015 | 0.017 | 0.624 | 238 | tags=42%, list=18%, signal=50% |
| 53 | LARSSON\_STATE\_3 |  | 28 | -0.42 | -1.77 | 0.013 | 0.021 | 0.699 | 182 | tags=32%, list=14%, signal=36% |
| 54 | SINGH\_SOX2\_DOWNREGULATED\_TRANSCRIPTS\_PMID\_28122245 |  | 19 | -0.46 | -1.76 | 0.023 | 0.022 | 0.730 | 121 | tags=26%, list=9%, signal=29% |
| 55 | GANDAL\_IBD\_UP\_PMID29439242 |  | 16 | -0.49 | -1.76 | 0.018 | 0.022 | 0.734 | 108 | tags=31%, list=8%, signal=34% |
| 56 | VANGRONINGEN\_MES |  | 85 | -0.32 | -1.72 | 0.008 | 0.027 | 0.805 | 249 | tags=33%, list=19%, signal=38% |
| 57 | ALMSTEDT\_VGR\_DIFF |  | 152 | -0.30 | -1.72 | 0.003 | 0.029 | 0.835 | 284 | tags=34%, list=21%, signal=38% |
| 58 | IVY\_IT |  | 15 | -0.48 | -1.69 | 0.031 | 0.035 | 0.888 | 242 | tags=47%, list=18%, signal=56% |
| 59 | IVY\_CTPAN |  | 25 | -0.42 | -1.68 | 0.019 | 0.036 | 0.893 | 107 | tags=32%, list=8%, signal=34% |
| 60 | LARSSON\_STATE\_2 |  | 22 | -0.42 | -1.62 | 0.040 | 0.055 | 0.972 | 158 | tags=32%, list=12%, signal=36% |
| 61 | ALLEN\_112\_ASTROL1FGFR3MT1G |  | 15 | -0.47 | -1.60 | 0.044 | 0.062 | 0.981 | 119 | tags=33%, list=9%, signal=36% |
| 62 | RICHARDS\_NOWAKOWSKI\_SCIENCE\_2017\_MGE\_RG2\_UPREG |  | 148 | -0.28 | -1.59 | 0.006 | 0.062 | 0.982 | 298 | tags=34%, list=23%, signal=40% |
| 63 | GANDAL\_BIPOLAR\_UP\_PMID29439242 |  | 26 | -0.39 | -1.58 | 0.055 | 0.066 | 0.985 | 280 | tags=42%, list=21%, signal=53% |
| 64 | FELDMAN\_2020\_LOWRNA |  | 248 | -0.25 | -1.51 | 0.011 | 0.103 | 0.999 | 227 | tags=22%, list=17%, signal=22% |
| 65 | RICHARDS\_CAHOY\_JNEUROSCI\_2012\_OPC |  | 471 | -0.24 | -1.46 | 0.001 | 0.132 | 1.000 | 574 | tags=50%, list=43%, signal=57% |
| 66 | OLSEN\_ADRENERGIC |  | 20 | -0.38 | -1.45 | 0.094 | 0.141 | 1.000 | 106 | tags=20%, list=8%, signal=21% |
| 67 | ALMSTEDT\_11Q\_MSIGDB |  | 45 | -0.30 | -1.39 | 0.110 | 0.193 | 1.000 | 448 | tags=49%, list=34%, signal=71% |
| 68 | ALMSTEDT\_17Q\_MSIGDB |  | 43 | -0.30 | -1.38 | 0.127 | 0.196 | 1.000 | 25 | tags=9%, list=2%, signal=9% |
| 69 | RICHARDS\_NOWAKOWSKI\_SCIENCE\_2017\_EN\_PFC3\_UPREG |  | 59 | -0.28 | -1.37 | 0.130 | 0.206 | 1.000 | 733 | tags=75%, list=55%, signal=160% |
| 70 | VANGRONINGEN\_ADRN |  | 67 | -0.26 | -1.36 | 0.128 | 0.220 | 1.000 | 276 | tags=31%, list=21%, signal=38% |
| 71 | RICHARDS\_NOWAKOWSKI\_SCIENCE\_2017\_ASTROCYTE\_UPREG |  | 165 | -0.24 | -1.34 | 0.077 | 0.237 | 1.000 | 280 | tags=27%, list=21%, signal=30% |
| 72 | GAROFANO\_IAVARONE\_GPM\_SUBTYPE |  | 176 | -0.23 | -1.34 | 0.065 | 0.236 | 1.000 | 344 | tags=32%, list=26%, signal=37% |
| 73 | RICHARDS\_NOWAKOWSKI\_SCIENCE\_2017\_MURAL\_UPREG |  | 125 | -0.24 | -1.32 | 0.100 | 0.249 | 1.000 | 241 | tags=24%, list=18%, signal=27% |
| 74 | ALLEN\_111\_ASTROL1\_6FGFR3ETNPPL |  | 16 | -0.37 | -1.31 | 0.165 | 0.257 | 1.000 | 738 | tags=88%, list=56%, signal=196% |
| 75 | DOROSZKO\_U3013MG\_CLUSTER\_3 |  | 27 | -0.32 | -1.31 | 0.179 | 0.259 | 1.000 | 178 | tags=26%, list=13%, signal=29% |
| 76 | LARSSON\_STATE\_5 |  | 31 | -0.30 | -1.31 | 0.159 | 0.258 | 1.000 | 111 | tags=19%, list=8%, signal=21% |
| 77 | KLING\_GBM\_PHENO\_PMID\_27667176 |  | 30 | -0.31 | -1.30 | 0.163 | 0.270 | 1.000 | 145 | tags=23%, list=11%, signal=26% |
| 78 | RICHARDS\_VERHAAK\_CANCERCELL\_2010\_MESENCHYMAL |  | 20 | -0.34 | -1.28 | 0.191 | 0.285 | 1.000 | 545 | tags=65%, list=41%, signal=109% |
| 79 | TF\_CENSUS\_TAIPALE |  | 113 | -0.23 | -1.26 | 0.175 | 0.314 | 1.000 | 307 | tags=27%, list=23%, signal=33% |
| 80 | DOROSZKO\_U3054MG\_CLUSTER\_6 |  | 40 | -0.27 | -1.24 | 0.225 | 0.340 | 1.000 | 565 | tags=58%, list=43%, signal=97% |
| 81 | RICHARDS\_CAHOY\_JNEUROSCI\_2008\_ASTROCYTE |  | 297 | -0.21 | -1.23 | 0.110 | 0.345 | 1.000 | 571 | tags=49%, list=43%, signal=67% |
| 82 | NB\_KI\_CLUSTER23 |  | 49 | -0.25 | -1.20 | 0.252 | 0.386 | 1.000 | 791 | tags=78%, list=60%, signal=186% |
| 83 | RICHARDS\_NOWAKOWSKI\_SCIENCE\_2017\_IN\_CTX\_MGE1\_UPREG |  | 50 | -0.25 | -1.20 | 0.265 | 0.398 | 1.000 | 497 | tags=52%, list=38%, signal=80% |
| 84 | RICHARDS\_NOWAKOWSKI\_SCIENCE\_2017\_VRG\_UPREG |  | 134 | -0.21 | -1.16 | 0.270 | 0.453 | 1.000 | 224 | tags=23%, list=17%, signal=25% |
| 85 | RICHARDS\_ZHONG\_NATURE\_2018\_MICROGLIA\_UPREG |  | 43 | -0.24 | -1.11 | 0.338 | 0.545 | 1.000 | 269 | tags=26%, list=20%, signal=31% |
| 86 | RICHARDS\_NOWAKOWSKI\_SCIENCE\_2017\_ORG\_UPREG |  | 311 | -0.18 | -1.10 | 0.317 | 0.560 | 1.000 | 227 | tags=20%, list=17%, signal=18% |
| 87 | WANG\_CLASSICAL\_PMID\_28697342 |  | 17 | -0.30 | -1.08 | 0.385 | 0.594 | 1.000 | 693 | tags=76%, list=52%, signal=159% |
| 88 | RICHARDS\_NOWAKOWSKI\_SCIENCE\_2017\_MICROGLIA\_UPREG |  | 114 | -0.20 | -1.07 | 0.390 | 0.609 | 1.000 | 293 | tags=25%, list=22%, signal=29% |
| 89 | RICHARDS\_NOWAKOWSKI\_SCIENCE\_2017\_EN\_V1\_2\_UPREG |  | 120 | -0.19 | -1.06 | 0.400 | 0.621 | 1.000 | 354 | tags=32%, list=27%, signal=39% |
| 90 | RICHARDS\_CAHOY\_JNEUROSCI\_2014\_MOG\_POS |  | 99 | -0.20 | -1.05 | 0.431 | 0.640 | 1.000 | 237 | tags=21%, list=18%, signal=24% |
| 91 | RICHARDS\_NOWAKOWSKI\_SCIENCE\_2017\_EN\_PFC1\_UPREG |  | 171 | -0.18 | -1.05 | 0.437 | 0.635 | 1.000 | 586 | tags=50%, list=44%, signal=79% |
| 92 | RICHARDS\_NOWAKOWSKI\_SCIENCE\_2017\_CHOROID\_UPREG |  | 105 | -0.19 | -1.04 | 0.418 | 0.643 | 1.000 | 522 | tags=47%, list=40%, signal=71% |
| 93 | RICHARDS\_CAHOY\_JNEUROSCI\_2013\_OL\_MYEL |  | 147 | -0.18 | -1.03 | 0.439 | 0.661 | 1.000 | 732 | tags=64%, list=55%, signal=127% |
| 94 | IVY\_CTMVP |  | 17 | -0.28 | -1.01 | 0.459 | 0.680 | 1.000 | 353 | tags=41%, list=27%, signal=55% |
| 95 | RICHARDS\_NOWAKOWSKI\_SCIENCE\_2017\_TRG\_UPREG |  | 253 | -0.17 | -1.01 | 0.479 | 0.685 | 1.000 | 227 | tags=20%, list=17%, signal=20% |
| 96 | RICHARDS\_NOWAKOWSKI\_SCIENCE\_2017\_EN\_PFC2\_UPREG |  | 163 | -0.17 | -0.98 | 0.533 | 0.734 | 1.000 | 582 | tags=49%, list=44%, signal=77% |
| 97 | RICHARDS\_CAHOY\_JNEUROSCI\_2009\_NEURON |  | 135 | -0.17 | -0.97 | 0.540 | 0.749 | 1.000 | 388 | tags=32%, list=29%, signal=40% |
| 98 | NB\_KI\_CLUSTER13 |  | 20 | -0.26 | -0.97 | 0.514 | 0.744 | 1.000 | 588 | tags=60%, list=45%, signal=106% |
| 99 | RICHARDS\_NOWAKOWSKI\_SCIENCE\_2017\_EN\_V1\_1\_UPREG |  | 167 | -0.16 | -0.94 | 0.610 | 0.792 | 1.000 | 582 | tags=49%, list=44%, signal=76% |
| 100 | NB\_KI\_CLUSTER2 |  | 52 | -0.19 | -0.93 | 0.556 | 0.792 | 1.000 | 744 | tags=69%, list=56%, signal=152% |
| 101 | RICHARDS\_NOWAKOWSKI\_SCIENCE\_2017\_ENDOTHELIAL\_UPREG |  | 216 | -0.16 | -0.92 | 0.633 | 0.809 | 1.000 | 332 | tags=26%, list=25%, signal=29% |
| 102 | RICHARDS\_NOWAKOWSKI\_SCIENCE\_2017\_EN\_V1\_3\_UPREG |  | 162 | -0.16 | -0.91 | 0.649 | 0.816 | 1.000 | 578 | tags=47%, list=44%, signal=73% |
| 103 | RICHARDS\_ZHONG\_NATURE\_2018\_OPC\_UPREG |  | 29 | -0.21 | -0.90 | 0.579 | 0.830 | 1.000 | 195 | tags=21%, list=15%, signal=24% |
| 104 | GAROFANO\_IAVARONE\_NEU\_SUBTYPE |  | 37 | -0.20 | -0.89 | 0.604 | 0.846 | 1.000 | 531 | tags=49%, list=40%, signal=79% |
| 105 | DOROSZKO\_U3180MG\_CLUSTER\_4 |  | 45 | -0.18 | -0.88 | 0.611 | 0.847 | 1.000 | 340 | tags=31%, list=26%, signal=40% |
| 106 | RICHARDS\_CAHOY\_JNEUROSCI\_2011\_ASTRO\_MATURE |  | 107 | -0.16 | -0.87 | 0.677 | 0.857 | 1.000 | 776 | tags=64%, list=59%, signal=144% |
| 107 | RICHARDS\_NOWAKOWSKI\_SCIENCE\_2017\_IN\_CTX\_CGE2\_UPREG |  | 48 | -0.18 | -0.86 | 0.645 | 0.866 | 1.000 | 484 | tags=42%, list=37%, signal=63% |
| 108 | GAROFANO\_IAVARONE\_MTC\_SUBTYPE |  | 42 | -0.18 | -0.84 | 0.651 | 0.889 | 1.000 | 564 | tags=50%, list=43%, signal=84% |
| 109 | NB\_KI\_CLUSTER17 |  | 25 | -0.21 | -0.84 | 0.640 | 0.886 | 1.000 | 280 | tags=28%, list=21%, signal=35% |
| 110 | DOROSZKO\_U3180MG\_CLUSTER\_2 |  | 96 | -0.16 | -0.83 | 0.702 | 0.886 | 1.000 | 384 | tags=29%, list=29%, signal=38% |
| 111 | RICHARDS\_NOWAKOWSKI\_SCIENCE\_2017\_U1\_UPREG |  | 204 | -0.14 | -0.82 | 0.777 | 0.901 | 1.000 | 233 | tags=18%, list=18%, signal=18% |
| 112 | RICHARDS\_NOWAKOWSKI\_SCIENCE\_2017\_IN\_STR\_UPREG |  | 26 | -0.20 | -0.81 | 0.689 | 0.901 | 1.000 | 666 | tags=62%, list=50%, signal=122% |
| 113 | RICHARDS\_NOWAKOWSKI\_SCIENCE\_2017\_MGE\_RG1\_UPREG |  | 89 | -0.15 | -0.81 | 0.748 | 0.894 | 1.000 | 315 | tags=25%, list=24%, signal=30% |
| 114 | NB\_KI\_CLUSTER4 |  | 25 | -0.20 | -0.80 | 0.716 | 0.907 | 1.000 | 384 | tags=36%, list=29%, signal=50% |
| 115 | NB\_KI\_CLUSTER16 |  | 20 | -0.21 | -0.79 | 0.690 | 0.905 | 1.000 | 373 | tags=35%, list=28%, signal=48% |
| 116 | RICHARDS\_CAHOY\_JNEUROSCI\_2015\_ASTRO\_IN\_VIVO |  | 246 | -0.13 | -0.79 | 0.828 | 0.904 | 1.000 | 688 | tags=55%, list=52%, signal=93% |
| 117 | RICHARDS\_VERHAAK\_CANCERCELL\_2010\_PRONEURAL |  | 33 | -0.18 | -0.79 | 0.735 | 0.897 | 1.000 | 273 | tags=27%, list=21%, signal=34% |
| 118 | RICHARDS\_NOWAKOWSKI\_SCIENCE\_2017\_IN\_CTX\_MGE2\_UPREG |  | 48 | -0.16 | -0.79 | 0.736 | 0.891 | 1.000 | 585 | tags=50%, list=44%, signal=86% |
| 119 | DOROSZKO\_U3054MG\_CLUSTER\_5 |  | 23 | -0.20 | -0.77 | 0.728 | 0.899 | 1.000 | 145 | tags=17%, list=11%, signal=19% |
| 120 | RICHARDS\_CAHOY\_JNEUROSCI\_2008\_OLIGODENDROCYTE |  | 171 | -0.13 | -0.76 | 0.843 | 0.902 | 1.000 | 710 | tags=58%, list=54%, signal=109% |
| 121 | DOROSZKO\_U3054MG\_CLUSTER\_3 |  | 47 | -0.16 | -0.73 | 0.792 | 0.928 | 1.000 | 872 | tags=77%, list=66%, signal=217% |
| 122 | NB\_KI\_CLUSTER14 |  | 35 | -0.16 | -0.73 | 0.788 | 0.925 | 1.000 | 45 | tags=9%, list=3%, signal=9% |
| 123 | RICHARDS\_VERHAAK\_CANCERCELL\_2010\_CLASSICAL |  | 38 | -0.16 | -0.72 | 0.791 | 0.925 | 1.000 | 875 | tags=76%, list=66%, signal=220% |
| 124 | DOROSZKO\_U3054MG\_CLUSTER\_10 |  | 40 | -0.16 | -0.72 | 0.782 | 0.918 | 1.000 | 373 | tags=33%, list=28%, signal=44% |
| 125 | GANDAL\_ALCOHOLADDICTION\_DN\_PMID29439242 |  | 16 | -0.21 | -0.71 | 0.814 | 0.926 | 1.000 | 225 | tags=25%, list=17%, signal=30% |
| 126 | KRONA\_ROSEN\_WHITE\_MATTER\_INVASION |  | 46 | -0.14 | -0.66 | 0.865 | 0.964 | 1.000 | 562 | tags=46%, list=43%, signal=77% |
| 127 | RICHARDS\_NOWAKOWSKI\_SCIENCE\_2017\_NEN\_EARLY2\_UPREG |  | 76 | -0.12 | -0.65 | 0.877 | 0.967 | 1.000 | 228 | tags=18%, list=17%, signal=21% |
| 128 | RICHARDS\_NOWAKOWSKI\_SCIENCE\_2017\_GLYC\_UPREG |  | 93 | -0.11 | -0.62 | 0.900 | 0.978 | 1.000 | 53 | tags=5%, list=4%, signal=5% |
| 129 | NB\_KI\_CLUSTER3 |  | 29 | -0.14 | -0.59 | 0.904 | 0.991 | 1.000 | 410 | tags=38%, list=31%, signal=54% |
| 130 | NB\_KI\_CLUSTER8 |  | 46 | -0.12 | -0.57 | 0.905 | 0.994 | 1.000 | 670 | tags=57%, list=51%, signal=111% |
| 131 | RICHARDS\_NOWAKOWSKI\_SCIENCE\_2017\_MGE\_IPC3\_UPREG |  | 31 | -0.13 | -0.54 | 0.922 | 1.000 | 1.000 | 469 | tags=35%, list=36%, signal=54% |
| 132 | RICHARDS\_NOWAKOWSKI\_SCIENCE\_2017\_U3\_UPREG |  | 125 | -0.09 | -0.50 | 0.969 | 1.000 | 1.000 | 377 | tags=26%, list=29%, signal=33% |
| 133 | RICHARDS\_INHOUSE\_BULKRNASEQ\_2019\_DEVELOPMENTALGSC |  | 266 | -0.08 | -0.50 | 0.990 | 1.000 | 1.000 | 579 | tags=41%, list=44%, signal=58% |
| 134 | RICHARDS\_NOWAKOWSKI\_SCIENCE\_2017\_NEN\_LATE\_UPREG |  | 61 | -0.10 | -0.50 | 0.962 | 1.000 | 1.000 | 733 | tags=61%, list=55%, signal=130% |
| 135 | RICHARDS\_NOWAKOWSKI\_SCIENCE\_2017\_NIN1\_UPREG |  | 38 | -0.10 | -0.47 | 0.977 | 1.000 | 1.000 | 84 | tags=8%, list=6%, signal=8% |
| 136 | RICHARDS\_NOWAKOWSKI\_SCIENCE\_2017\_NEN\_EARLY1\_UPREG |  | 86 | -0.09 | -0.46 | 0.982 | 0.997 | 1.000 | 186 | tags=13%, list=14%, signal=14% |
| 137 | RICHARDS\_NOWAKOWSKI\_SCIENCE\_2017\_OPC\_UPREG |  | 90 | -0.08 | -0.44 | 0.989 | 0.994 | 1.000 | 107 | tags=8%, list=8%, signal=8% |
| 138 | DOROSZKO\_U3054MG\_CLUSTER\_9 |  | 56 | -0.09 | -0.43 | 0.987 | 0.990 | 1.000 | 331 | tags=21%, list=25%, signal=27% |
Table: Gene sets enriched in phenotype **na **[plain text format]****
